# Vitrectomy vs. Spontaneous Closure for Traumatic Macular Hole: A Systematic Review and Meta-Analysis

**DOI:** 10.3389/fmed.2021.735968

**Published:** 2021-12-23

**Authors:** Qi Zhou, Haoyue Feng, Hongbin Lv, Zhongmei Fu, Yuyu Xue, Hejiang Ye

**Affiliations:** ^1^Eye School, Chengdu University of Traditional Chinese Medicine, Chengdu, China; ^2^Department of Ophthalmology, Affiliated Hospital of Chengdu University of Traditional Chinese Medicine, Chengdu, China; ^3^Department of Ophthalmology, Affiliated Hospital of Southwest Medical University, Luzhou, China

**Keywords:** traumatic macular hole, closure rate, visual acuity improvement, vitrectomy, spontaneous closure, meta-analysis

## Abstract

**Objective:** This systematic review and meta-analysis aimed to determine the traumatic macular hole (TMH) closure rate and visual acuity (VA) improvement rate by comparing two treatment methods for TMH: vitrectomy and observation for spontaneous closure.

**Methods:** PubMed, Cochrane, Web of Science Library, Embase, CNKI, Wanfang, VIP, and Sino Med were systematically searched from their inception to June 10, 2021. Studies in the surgery group (*n* = 32) and studies in the observation group (*n* = 12) were meta-analyzed. The primary outcomes were the TMH closure and VA improvement rates in the surgery and observation groups. The secondary outcomes were best-corrected visual acuity (BCVA) improvement in the surgery group. Stata software (version 15.1) was used for the analyses.

**Results:** Thirty-six studies that included 1,009 eyes were selected for this meta-analysis, among which 33 were retrospective studies and 3 were prospective studies. The meta-analysis showed that the random-model pooled event rate for TMH closure was 0.37 (95% confidence interval [CI], 0.26–0.48) in the observation group, while it was 0.9 (95% CI, 0.85–0.94) in the surgery group. The fixed-model pooled event rate for VA improvement was 0.39 (95% CI, 0.33–0.45) in the observation group, while the random-model pooled event rate of VA improvement for the surgery group was 0.72 (95% CI, 0.63–0.80). The pooled event rate for BCVA improvement in the surgery group was 0.39 (95% CI, 0.33–0.46).

**Conclusions:** This meta-analysis suggests that TMH hole closure and VA improvement rates in the surgery group were significantly higher than those in the observation group. Vitrectomy is an effective method for treating TMH. However, further randomized controlled trials (RCTs) are required to evaluate the efficacy and safety of surgery and observation for TMH treatment.

**Systematic Review Registration:** https://www.crd.york.ac.uk/PROSPERO/#recordDetails, identifier: CRD42021276684.

## Introduction

A macular hole (MH) is a defined as a full-thickness defect of the neuroretina in the macular foveal area. Traumatic macular holes (TMH) represent approximately 10% of MHs and may result in permanent significant vision loss (1). TMH is often found in young men, as the condition it is frequently associated with sport- and work-related accidents (2). The functional outcomes are often unclear because of the accompanying trauma-induced retinal pathologies, such as vitreous hemorrhage, retinal detachment, retinal hemorrhage, choroidal fracture, subretinal choroidal neovascularization, and fibrosis.

However, the posttraumatic approach is controversial. To date, no clinical guidelines have been established for this vision-threatening disease. Treatment includes vitrectomy surgery and observation, as well as spontaneous closure (1). Vitrectomy has been reported to improve anatomical and visual outcomes in eyes with TMH (3, 4). Currently, surgical techniques include removing the posterior vitreous cortex and epiretinal membranes, with or without internal limiting membrane (ILM) peeling, and intraocular gas or silicone oil tamponade. Various adjuvant therapies, including transforming growth factor-beta (TGF-β), biological tissue adhesives, and platelet concentrate, have been investigated with varying degrees of success (5, 6). However, there are many unanswered questions about the necessity of surgery because spontaneous hole closure has been commonly reported (7). Many studies have reported that spontaneous closure usually occurs between 1 and 6 months after the trauma incident (8, 9). While a number of studies have discussed the anatomical and visual outcomes of surgery and observation on TMH, a previous systemic review and meta-analysis, which included only 10 studies, lacked sufficient detail (10). Therefore, in this systematic review and meta-analysis, we systematically and statistically determined the improvement rates with TMH closure and visual acuity (VA) by comparing the two methods of treating TMH.

## Materials and Methods

This systematic review and meta-analysis was performed according to the Meta-analyses Of Observational Studies in Epidemiology (MOOSE) guidelines (11). Ethical approval was not necessary for the study as it used published data. Four databases in English, including PubMed, Cochrane, Web of Science Library, and Embase, and four databases in Chinese, including CNKI, Wanfang, VIP, and Sino Med, were searched from their inception to June 10, 2021. Google Scholar and Baidu Scholar were also searched to find studies missing in those databases. A manual search was conducted to identify published studies. In the case of unpublished studies, the database was searched for their abstracts, and their authors were also contacted. EndNote was used to merge retrieved citations and eliminate duplications.

Two independent researchers (QZ and HYF) separately assessed the eligibility, extracted the data, and assessed the quality of the included studies, and a third author (HJY) determined the final criteria for any inconsistencies.

### Search Strategy

The search strategy included the following search terms: “retinal perforations,” “retinal hole,” “retinal tear,” “retinal break,” “macular hole,” “traumatic macular hole,” “vitrectomy,” “pars plana vitrectomy,” “surgical management,” “observation,” “treatment,” and “spontaneous closure.” The search terms are shown in the Supplementary Material.

### Inclusion Criteria

Articles were included if they (1) included studies on patients with TMH; (2) used closure rate and VA improvement rate as the treatment endpoints; (3) provided clinical statistics on age, sex, best-corrected visual acuity (BCVA) expressed in the logarithm of the minimal angle of resolution (logMAR), MH size, follow-up data, operation, interval from injury to surgery, TMH closure rate, and VA improvement rate; and (4) were published in Chinese or English full text.

### Exclusion Criteria

Articles were excluded if they (1) reported duplicated or overlapping data; (2) were designed as “reviews,” “case reports,” “letters” or “conference articles” with no data to extract; (3) focused on patients diagnosed with idiopathic MH, myopic MH, or TMH with retinal detachment; and (4) were not published in Chinese or English full text.

### Data Extraction

According to the inclusion and exclusion criteria, two researchers (QZ and HYF) independently read the full text of the articles and selected the qualified studies. The following information was extracted from eligible studies: first author and publication year, study design, country, follow-up time, sex, number of participants, BCVA logMAR before and after TMH closure, size of TMH (μm), closure rate, and VA improvement rate. For the surgery group patients, data on the interval from the injury to the surgery were extracted. For the observation group patients, data on the time of hole closure were extracted.

### Quality Assessment

To accurately evaluate the methodological quality of eligible studies, two researchers independently used the Newcastle-Ottawa Scale, which is a nine-point system including participant selection (0–4 points), comparability (0–2 points), and exposure (0–3 points) (12). Scores of 0–3, 4–6, and 7–9 points were regarded as low, moderate, and high quality, respectively. All included studies were identified to be of moderate or high methodological quality (Supplementary Material).

### Statistical Analysis

The primary outcomes were TMH closure rate and VA improvement rate in both groups. BCVA improvement in the surgery group was defined as a secondary outcome.

Continuous data are presented as mean ± standard deviation. The descriptive statistics (BCVA logMAR improvement, TMH closure, and VA improvement rate) were analyzed with a 95% confidence interval (CI). The Cochran Q test and *I*^2^ statistic were used to identify heterogeneity among the studies. When there was no significant heterogeneity (*I*^2^ < 50% or *P* > 0.05), we applied a fixed-effect model to estimate the pooled effect size; otherwise, a random-effect model was employed. Funnel plots were used to detect potential publication bias. A sensitivity analysis was performed to test the robustness of the analysis. A subgroup analysis was conducted to explore the potential heterogeneity among patients in the surgery according to the different surgical procedures. All data synthesis and analysis were performed using Stata version 15.1.

## Results

### Literature Search and Study Selection

**Figure 1:**
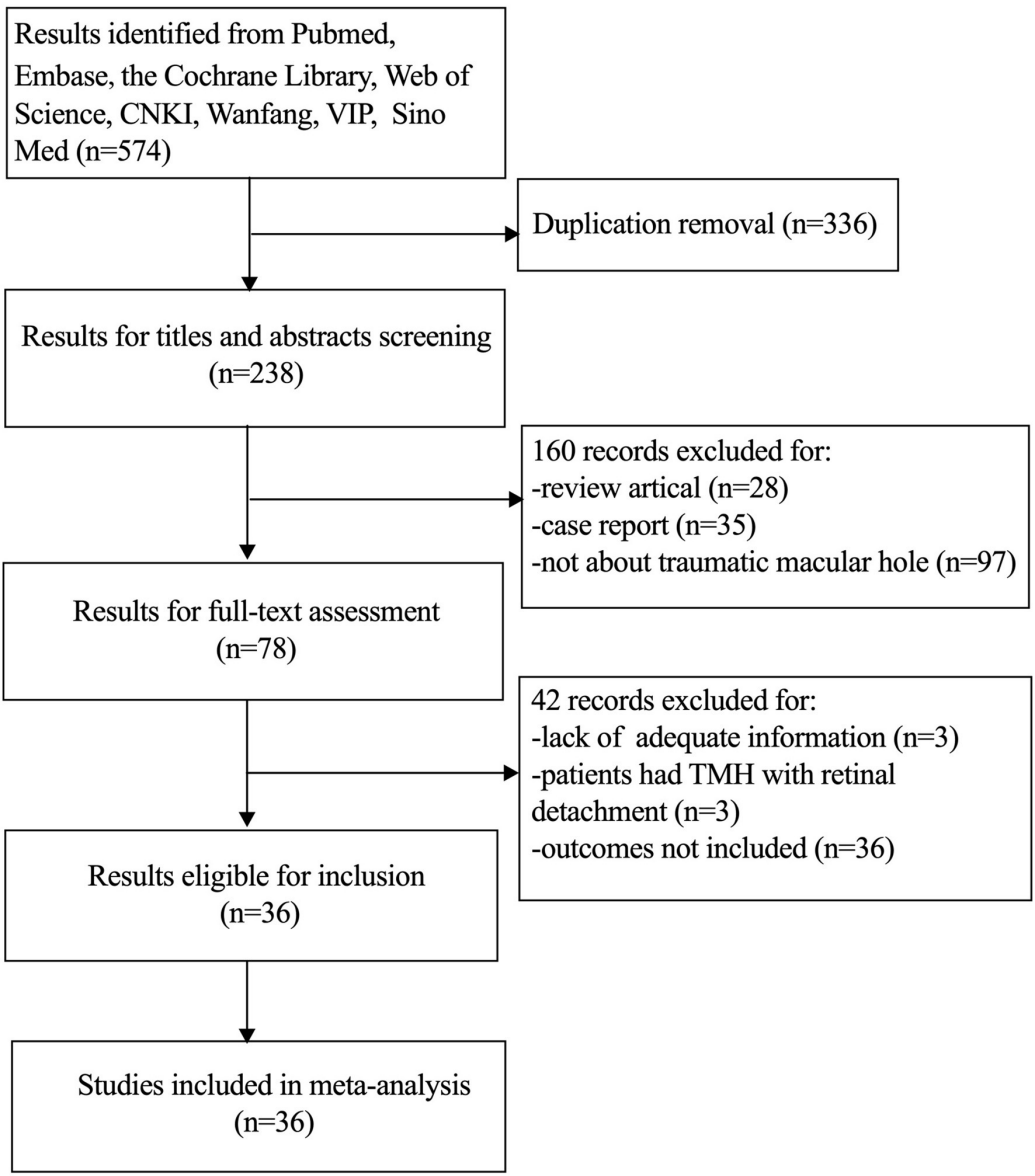
Flow chart of the study selection (through June 10, 2021).

A total of 574 records were identified with the initial search strategy. After removing 336 duplicates, 238 studies were assessed by title and abstract. Thirty-six studies, including 33 retrospective studies and three prospective studies, were selected for our meta-analysis according to the inclusion and exclusion criteria. The details of the search strategy are shown in Figure 1.

### Characteristics of the Study Samples

Thirty-two studies, as shown in Table 1, reported data on patients (*n* = 734) who underwent vitrectomy. For the surgery group patients, vitrectomies were performed using adjunctive therapies, including ILM peeling or flap, platelet concentrate or TGF-β, and gas or silicon oil tamponade. Most of the patients were males, with a similar proportion in both groups. The mean age of the surgery group patients was 26.95 years (*n* = 671; range, 1–69 years). The mean follow-up time was 10.41 ± 6.48 months (*n* = 662; median, 12; range, 3–45 months). The interval from injury to surgery ranged from 1 week to 120 months, and the average size of the TMH was 628.84 μm (*n* = 358; range, 64–1,588 μm). The mean preoperative and postoperative BCVA were 0.87 logMAR (*n* = 247) and 0.48 logMAR (*n* = 247), respectively. The pooled event rate for BCVA improvement in the surgery group was 0.39 (95% CI, 0.33–0.46).

**Table 1:** Study design and baseline patient characteristics of the surgery group studies.

**References**	**Design**	**Country**	**Follow-up (months)**	**No. of eyes**	**Mean age (years)**	**Gender** **(Male/ Female)**	**BCVA±SD logMAR (pre/post)**	**Size of Macular hole (μm)**	**Interval from injury to surgery**	**Operation**	**Closure Rate**	**VA improvement Rate**
Kunikata et al. (3)	Retrospective	Japan	12.9	18	18.3	15/3	0.65 ± 0.08/0.21 ± 0.07	312.5 ± 170.8	71.7 ± 44.2 days	PPV + ILM peeling or ILM flap + SF6	0.9444	1.0000
Chang et al. (13)	Prospective	China	3.56 ± 1.32	32	31.02 ± 5. 98	22/10	0.59 ± 0.12/0.14 ± 0.06	650.28 ± 34.19	1–4 months	PPV + ILM Peeling	0.9063	0.9375
Ghoraba et al. (14)	Retrospective	Egypt	37 ± 45	28	21.4 ± 13	23/5	NA	757 ± 221	9 ± 23.5 months	PPV + ILM peeling	0.7500	NA
Chen et al. (7)	Prospective	China	6	25	31.0 ± 12.5	22/3	1.00 ± 0.35/0.56 ± 0.36	512.4 ± 315.1	20.8 ± 8.8 days	PPV + ILM peeling + C3F8/air filling	1.0000	0.8800
Fan et al. (15)	Retrospective	China	3	33	37.02 ± 1.35	17/16	NA	NA	NA	PPV	0.7879	0.4242
Tang et al. (16)	Retrospective	Australian and New Zealand	12	23	43.2	NA	NA	374	117 days	PPV + ILM peeling (21 cases) + C3F8/SF6	0.9130	0.4783
Li et al. (17)	Retrospective	China	12	25	28.5 ± 10.1	NA	NA	281.3 ± 111.3	NA	PPV + ILM peeling+air	0.8000	0.2800
Li et al. (17)	Retrospective	China	12	28	26.1 ± 12.9	NA	NA	397.6 ± 98.2	NA	PPV + ILM peeling + C3F8	0.8214	0.3214
Fu et al. (18)	Retrospective	China	4.6 ± 0.5	30	36.4 ± 3.7	NA	0.12 ± 0.06/NA	648.5 ± 105.3	8.5 ± 5.7 days	PPV + ILM peeling + C3F8	0.8667	0.5000
Li et al. (19)	Retrospective	China	10	16	12–45	16/0	0.07 ± 0.01/0.33 ± 0.02	477 ± 183	NA	PPV + ILM peeling + air	0.8125	0.9375
Browne et al. (20)	Retrospective	Egypt	6	16	29.95 ± 9.98	14/2	1.1 ± 0.2/0.2 ± 0.13	401.44 ± 34.8	NA	PPV + ILM peeling + C3F8	0.9375	NA
Brennan et al. (21)	Retrospective	Switzerland	12	13	14.15 ± 2.882	10/3	0.91 ± 0.43/0.50 ± 0.17	NA	5.38 ± 3.5 months	PPV + ILM peeling + C3F8	0.9231	0.9231
Li et al. (22)	Retrospective	China	12	34	34.1 ± 7.4	NA	0.12 ± 0.07/NA	653.6 ± 123.9	40.8 ± 20.6 days	PPV + ILM peeling + C3F8	0.7059	0.3824
Zhu et al. (23)	Retrospective	China	6	28	29.01 ± 7.33	22/6	0.086 ± 0.101/0.202 ± 0.171	NA	NA	PPV + ILM peeling + C2F6	0.8571	0.6786
Abou Shousha et al. (24)	Prospective	Egypt	9	12	23.25 ± 14.11	8/4	NA	696 ± 445	3.75 ± 1.06 months	PPV + ILM flap + SF6	1.0000	0.9167
Chen et al. (25)	Retrospective	China	4–12	11	26.36 ± 8.43	8/3	NA	NA	4–14 months	PPV + ILM peeling + air	0.6364	0.4545
Yuan et al. (26)	Retrospective	China	12	26	32.4 ± 9.7	NA	0.13 ± 0.07/0.15 ± 0.07	643.3 ± 125	NA	PPV + ILM peeling + C3F8	0.6923	0.2692
Tian et al. (27)	Retrospective	China	12	10	44.6	6/4	NA	607.13	NA	PPV + ILM peeling + intraocular tamponade	0.8000	0.5000
Hou et al. (28)	Retrospective	China	1–27	54	27.2 ± 12.4	48/6	1.06 ± 0.39/0.84 ± 0.43	598 ± 227	1–156 months	PPV + ILM peeling + C3F8/SF6/C2F6 + platelet concentrate	0.8889	0.5185
Wan et al. (29)	Retrospective	China	6–14	24	NA	22/2	NA	623 ± 303	4–24 months	PPV + ILM peeling + C3F8	0.9167	0.7083
Ghoraba et al. (30)	Retrospective	Egypt	14.46 ± 3.43	13	26.54 ± 5.68	9/4	0.061/0.433	NA	NA	PPV+ILM peeling+C3F8	0.9231	NA
Qu et al. (31)	Retrospective	China	96 ± 131 days	95	26.6 ± 13.5	87/8	1.1 ± 0.45/0.83 ± 0.40	644.2 ± 270.5	9.8 ± 21.8 months	PPV + ILM peeling (90 cases) or not + C3F8/SF6/C2F6 + platelet concentrate (85 cases)	1.0000	0.7263
Ovali et al. (32)	Retrospective	Turkey	NA	14	40.4 ± 14.4	NA	NA	425	NA	PPV + ILM peeling + C3F8 (13 cases)/silicone-oil (1 case)	0.9286	0.8571
Chen et al. (33)	Retrospective	China	8	11	NA	NA				PPV + ILM peeling+air	0.6364	0.7273
Gong (34)	Retrospective	China	3–12	14	37.93 ± 12.92	12/12	0.058 ± 0.044/0.22 ± 0.21	628.79 ± 183.33	45.36 ± 45.24 days	PPV + ILM peeling	1.000	0.7143
Wu et al. (35)	Retrospective	America	12.5 ± 16.4	13	10	10/3	NA	NA	2.9 ± 2.0 months	Plasmin Enzyme-Assisted PPV + ILM peeling (3 cases) + C3F8/ silicone-oil	0.9231	0.9167
Ma et al. (36)	Retrospective	China	3–6	8	24.13	7/1	NA	NA	NA	PPV + ILM peeling + C3F8	0.8750	0.7500
Liu and Gong (37)	Retrospective	China	4-24	12	18–65	9/3	NA	NA	3–12 months	PPV + ILM peeling (3 cases) + C3F8/SF6	0.8333	0.7500
Kuhn et al. (38)	Retrospective	Hungary	14	17	26	15/2	NA	NA	2.5 months	PPV + ILM peeling + SF6	1.0000	0.9412
Johnson et al. (39)	Retrospective	America	11	25	23	20/5	NA	NA	NA	PPV + ILM peeling + C3F8	0.9600	0.8400
García-Arumí et al. (40)	Retrospective	Spain	13	14	19	11/3	NA	NA	1–6 weeks	PPV + platelet concentrate + SF6	0.9286	0.9286
Rubin et al. (41)	Retrospective	America	12.1	12	15	11/1	NA	NA	19 weeks	PPV + TGF-β + C3F8	0.9167	0.6667

*BCVA, best-corrected visual acuity; C2F6, hexafluoroethane; C3F8, perfluoropropane; SF6, sulfur hexafluoride; ILM, internal limiting membrane; logMAR, logarithm of the minimal angle of resolution; NA, not available; PPV, pars plana vitrectomy; VA, visual acuity*.

For the observation group, 12 studies and 275 patients were analyzed (Table 2). The average age of the observation group patients was 30.36 years (*n* = 157; range, 9–49 years), the mean follow-up time was 10.56 ± 5.15 months (*n* = 275, median, 12; range, 3–48 months), and the average size of the TMH was 561.10 μm (*n* = 93; range, 553.6–681.4 μm). The percentage of patients who achieved TMH closure in <6 months was > 80%.

**Table 2:** Study design and baseline patient characteristics of observation group studies.

**References**	**Design**	**Country**	**Follow-up** **(months)**	**No. of Patients**	**Mean age** **(years)**	**Gender** **(male/female)**	**BCVA ± SD logMAR (pre/post)**	**Size of macular hole (μm)**	**Time of hole closure**	**Closure Rate**	**VA improvement Rate**
Chen et al. (7)	Prospective	China	6	15	33.1 ± 11.6	14/1	1.11 ± 0.48/0.75 ± 0.4	423.2 ± 242.9	2.5 ± 1.6 months	0.6667	0.4667
Fan et al. (15)	Retrospective	China	3	30	37.28 ± 1.40	16/14	NA	NA	NA	0.5667	0.4333
Fu et al. (18)	Retrospective	China	4.6 ± 0.5	26	35.9 ± 3.4	NA	0.13 ± 0.08/NA	653.8 ± 94.7	12 months	0.4231	0.4615
Li et al. (22)	Retrospective	China	12	27	31.2 ± 5.5	19/8	0.13 ± 0.06/NA	632.5 ± 82.4	NA	0.4074	0.4074
Yuan et al. (26)	Retrospective	China	12	21	26.1 ± 10.0	NA	NA	490 ± 86.9	51.0 ± 12.6 days	0.3333	0.3333
Chen et al. (42)	Retrospective	China	6	27	26.2 ± 10.7	23/4	1.36 ± 0.74/1.01 ± 0.60	NA	NA	0.3704	0.3333
Tian et al. (27)	Retrospective	China	12	12	35	8/4	NA	NA	NA	0.6667	0.5000
Hou et al. (43)	Retrospective	China	16	30	32	27/3	NA	NA	NA	0.1000	0.4000
Chen et al. (33)	Retrospective	China	12	30	NA	NA	NA	NA	2.83 months	0.3000	0.3667
Li et al. (44)	Retrospective	China	14	28	30.1	25/3	NA	NA	4–5 months	0.1071	0.2857
Jin et al. (45)	Retrospective	China	20.72 ± 11.61	11	19.55 ± 8.18	10/1	NA	NA	NA	0.2727	0.2727
Yamashita et al. (46)	Retrospective	Japan	8.4	18	14.6	NA	NA	NA	1 week to 4 months	0.4444	0.4444

*BCVA, best-corrected visual acuity; logMAR, logarithm of the minimal angle of resolution; NA, not available; VA, visual acuity*.

### Pooled Rates of Closure and VA Improvement for the Surgery Group

**Figure 2:**
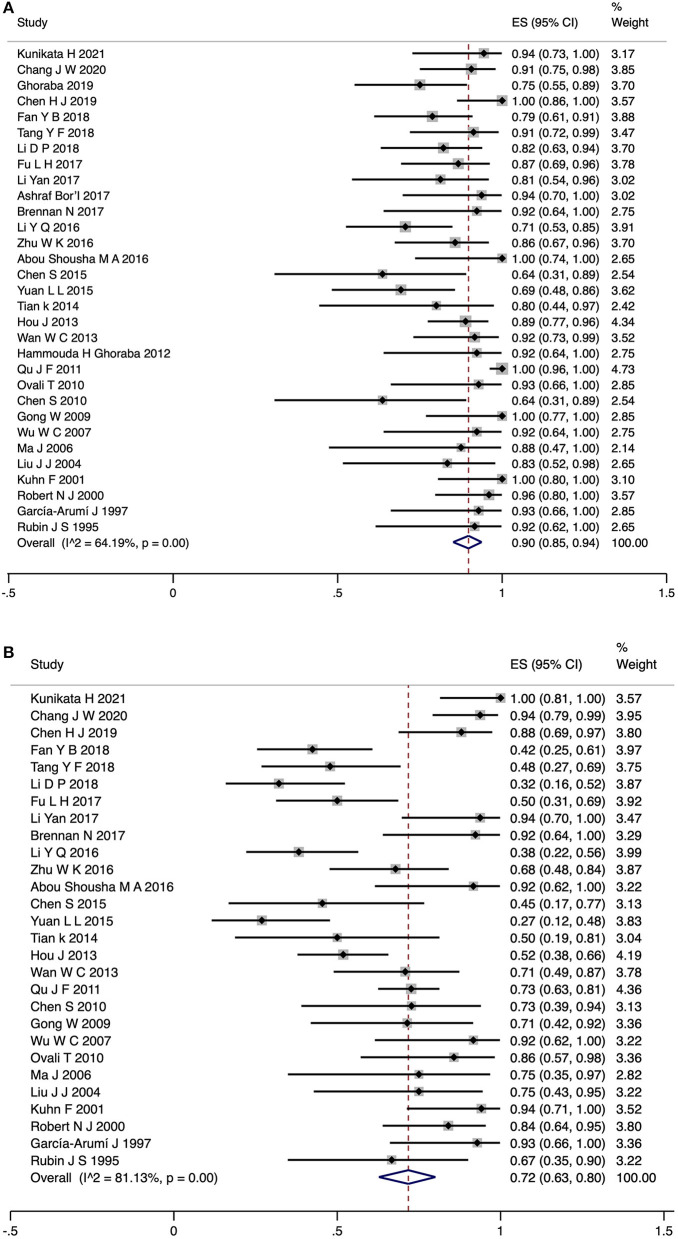
Forest plot for TMH closure rate **(A)** and VA improvement rate **(B)** of surgery group patients. TMH, traumatic macular hole; VA, visual acuity.

In the surgery group, the pooled rates of TMH closure and VA improvement were reported in 31 studies (709 eyes) and 28 studies (651 eyes), respectively. The random-model pooled rate for TMH closure was 0.9 (95% CI, 0.85–0.94, Figure 2A), while that for VA improvement was 0.72 (95% CI, 0.63–0.80). There was high heterogeneity between the studies (*I*^2^ = 64.19%, *P* < 0.05; *I*^2^ = 81.13%, *P* < 0.05, Figure 2B). The Funnel plots did not reveal evidence of publication bias (Supplementary Material). For the TMH closure rate, sensitivity analysis suggested that one study (31) may have been a potential source of heterogeneity (Supplementary Material). After excluding this study, the pooled rate of TMH closure in the remaining 30 studies was 0.89 (95% CI, 0.85–0.92, *I*^2^ = 40.10%, *P* < 0.05).

The results of the subgroup analyses are shown in Table 3. There was no significant difference in TMH closure rate between subgroups stratified by different types of operation. Statistically significant effects of the subgroups were identified for VA improvement rate and BCVA logMAR improvement (*P* < 0.05 for heterogeneity between groups).

**Table 3:** Subgroup analysis for outcomes.

	**No. of comparisons**	**Results**	***P* value for overall effect**	***I^**2**^***	***P* value for subgroup difference**
Type of operation	17	TMH closure rate (95% CI)		39.01%	0.05
PPV + ILM peeling + air	4	0.75 (0.63–0.86)	0.57	0%	
PPV + ILM peeling + C3F8/SF6/C2F6	13	0.88 (0.82–0.93)	0.09	37.22%	
Type of operation	14	VA improvement rate (95% CI)		81.85%	0.00
PPV + ILM peeling + air	4	0.61 (0.27–0.91)	0	86.10%	
PPV+ILM peeling+C3F8/SF6/C2F6	10	0.62 (0.48–0.76)	0	82.00%	
Type of operation	7	BCVA logMAR improvement (95% CI)		59.4%	0.022
PPV + ILM peeling + intraocular tamponade	5	0.45(0.41–0.48)	0.990	0%	
PPV + ILM peeling + platelet concentrate + intraocular tamponade	2	0.25(0.16–0.35)	0.022	0%	

*BCVA, best-corrected visual acuity; C3F8, perfluoropropane; SF6, sulfur hexafluoride; C2F6, hexafluoroethane; ILM, internal limiting membrane; logMAR, logarithm of the minimal angle of resolution; PPV, pars plana vitrectomy; TMH, traumatic macular hole; VA, visual acuity*.

The pooled event rate of TMH closure was higher in the pars plana vitrectomy (PPV) + ILM peeling + perfluoropropane (C3F8)/sulfur hexafluoride (SF6)/hexafluoroethane (C2F6) group (0.88, 95% CI: 0.82–0.93, *I*^2^ = 37.22%, Table 3) than in the PPV + ILM peeling + air group (0.75, 95% CI: 0.63–0.86, *I*^2^ = 0%, Table 3). However, there was still unexplained heterogeneity (*I*^2^ > 50%) between subgroups in the VA improvement rate, which the subgroup analysis could not completely explain. For BCVA logMAR improvement, the patients in the PPV+ILM peeling + platelet concentrate + intraocular tamponade group had a better BCVA improvement (0.25, 95% CI: 0.16–0.35, *I*^2^ = 0%, Table 3) than those in the PPV + ILM peeling + intraocular tamponade group (0.45, 95% CI: 0.41–0.48, *I*^2^ = 0%, Table 3). Meta-regression revealed that different types of operations affected the results of BCVA logMAR improvement.

### Pooled Rates of Closure and VA Improvement for the Observation Group

**Figure 3:**
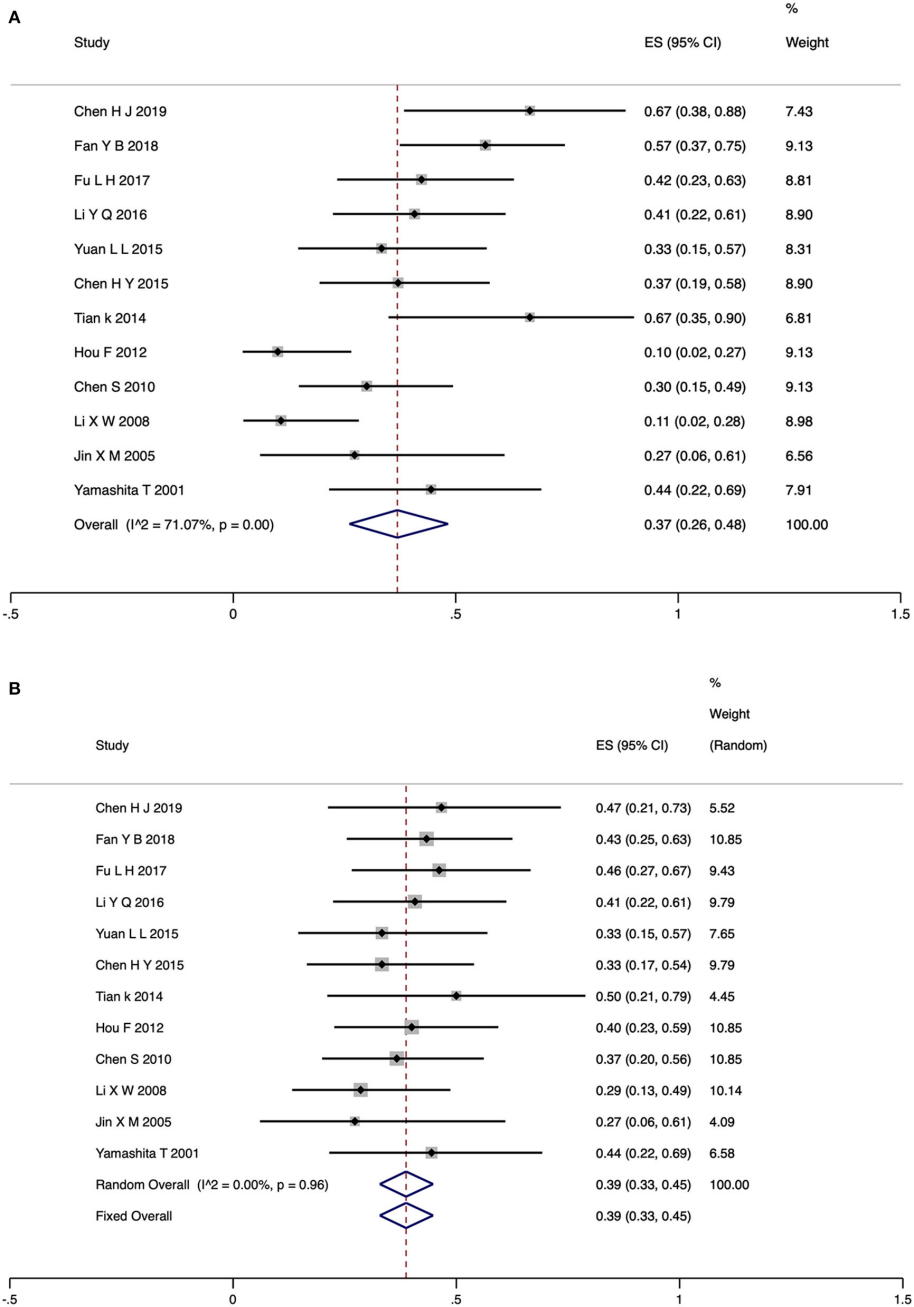
Forest plot for TMH closure rate **(A)** and VA improvement rate **(B)** of observation group patients. TMH, traumatic macular hole; VA, visual acuity.

For the observation group, the rate of TMH closure and VA improvement was reported in 12 studies (275 eyes). The random-model pooled rate for TMH closure was 0.37 (95% CI, 0.26–0.48). There was high heterogeneity among the studies (*I*^2^ = 71.07%, *P* < 0.05) (Figure 3A). The included studies were not randomized controlled trials (RCTs), which may be a potential source of heterogeneity. Publication bias was not assessed. Sensitivity analysis showed that the results were robust (Supplementary Material). The pooled event rate for VA improvement was 0.39 (95% CI, 0.33–0.45; fixed model) with no heterogeneity between the studies (*I*^2^ = 0.00%, *P* > 0.05) (Figure 3B).

### Adverse Effects

Mild vitreous hemorrhage was noted in one patient one day after vitrectomy surgery, which resolved within 1 week (40). Vitreoretinal surgery combined with the use of intraocular gases can result in elevated postoperative intraocular pressure (IOP) and cataract formation. Three studies reported increased IOP after surgery, but it was controlled within the normal range after medication (30, 37). In six studies, 24 patients developed cataract formation or acceleration during the follow-up period (17, 23, 29, 30, 37). After the operation, three patients from two studies developed retinal detachment (17, 31). One of the reasons for this was an improper surgical operation. None of the patients in these studies developed endophthalmitis. In the observation group, 17 eyes developed obvious hole enlargement and two eyes had retinal detachment (43, 44).

## Discussion

### Summary of the Main Results

We obtained several results by combining the existing evidence. First, although the studies included in our meta-analysis were of moderate or high methodological quality, there were no RCTs of TMH treatment, which may have led to a lack of convincing results. Second, the rates of TMH closure and VA improvement were significantly higher in the surgery group than in the observation group. This evidence may represent the best available support for treating patients with vitrectomy. Third, TMH patients were younger and mainly males, and over 80% of them showed closure with observation in <6 months. Raised IOP and cataracts are common postoperative complications, but these will not be severely adverse if immediate and proper treatment is adopted.

### Rate of Closure and VA Improvement According to Vitrectomy Surgery

In this systematic review, the TMH closure rate ranges from 0.63 to 1.0 with a pooled event rate of 0.90, while the VA improvement rate ranges from 0.28 to 1.0, with a pooled event rate of 0.72 in patients undergoing surgery. According to the study by Wang and Peng (47), the closure and VA improvement rates were 0.83 and 0.84, respectively, so that our results are similar to theirs, and showed that vitrectomy surgery seems to be a more effective method than observation for TMH treatment. The widespread use of optical coherence tomography (OCT) can offer further insight into the nature of TMH and shed light on the possible reasons for this Miller et al. (2) reported that an intact ellipsoid zone in closed holes tended to correlate with improved final visual acuity. A multicenter prospective comparative study showed that there were no significant differences in the length of the photoreceptor IS/OS junction (ellipsoid zone) defect and the final BCVA between the surgically closed cases and spontaneously closed cases, with 80% of the patients showing spontaneous hole closure within 3 months (7). Thus a 3-month observation period after injury may be an alternative modality for TMH management. Therefore, many researchers suggested that vitreous surgery should be carried out in 3 months to prevent severe photoreceptor damages. The closure rate was higher than the VA improvement rate in the surgery group, and the differences between anatomical and functional outcomes may be associated with different preoperative retinal pathologies and ocular complications (1). The study by Qu et al. (31), which reported a TMH closure rate of 1.0, may be a potential source of heterogeneity. The reason for the high closure rate may be associated with the use of adjunctive therapy (platelet concentrate).

The underlying mechanism of TMH formation is unclear. One type forms immediately after ocular trauma, with the foveal rupture causing acute vision loss. Another type may result from the development of macular edema and cysts, which may induce delayed-onset TMH formation. With the regression of macular edema, shrinkage and closure of the hole may occur. Glial cell proliferation and epiretinal membrane formation are often the causes of a persistent open hole. Therefore, vitrectomy with membrane peeling might be helpful and is a standard surgical procedure for treating TMH (6). Currently, PPV, ILM peeling or flap, and intraocular gas or silicone oil tamponade are the most commonly employed surgical procedures for TMH treatment (5). Ghoraba et al. concluded that gas tamponade is more successful than silicone oil tamponade for the anatomical closure and VA improvement of TMH (30). Intraocular gas tamponade is a crucial component of the surgical procedure for TMH repair. Higher rates of TMH closure were observed with C3F8, SF6, or C2F6 ocular tamponades, which could result from the extended amount of time the C3F8, SF6, or C2F6 lasts in the vitreous cavity. In this meta-analysis, the TMH closure rate and VA improvement rate in the C3F8/SF6/C2F6 tamponade group showed better outcomes than that in the air tamponade group.

Adjunctive therapies are often used together with surgery to accelerate hole closure (28, 40, 41). Rubin et al. used TGF-β2 in 12 eyes during vitrectomy and finally achieved a closure rate of 67% in eight eyes (41). Garca-Arum et al. found that the intraoperative application of platelet concentrate in combination with vitrectomy may help improve anatomic and visual outcomes (40). As shown by our meta-analysis, platelet concentrate was a potential factor that affected visual improvement. However, studies are unlikely to be designed to evaluate adjunctive therapies, which are also seldom implemented today.

### Spontaneous TMH Closure

TMH has been shown to close without any treatment, usually between 1 and 6 months after the trauma incident (8, 9). The closure and VA improvement rates were 0.37 and 0.39, respectively, similar to those reported in previous publications. In our meta-analysis, over 80% of the patients with TMH achieved closure within 6 months. The mechanism of spontaneous closure is of great interest. Why does TMH have a higher spontaneous closure rate than other types of MH? The fact that TMH patients are young and have a healthy vitreous gel and a firm vitreofoveal attachment may account for the high rate of spontaneous closure. Indeed, young age, small hole size, cystic edema at the edge of the MH, and no posterior vitreous detachment have been recognized as possible features affecting spontaneous closure (7, 48).

In addition, it should be noted that 17 eyes developed obvious hole enlargement and two eyes showed retinal detachment (43, 44). In five studies, 134 patients received supportive drugs (such as Sanqi Panax notoginseng for injection, compound anisodine hydrobromide injection, inosine tablets, or iodizedlecithin) to prepare the optic nerve and promote retinal microcirculation. However, experimental or clinical proof about their efficacy is lacking.

### Limitations

This meta-analysis has some limitations. First, high heterogeneity existed in some outcomes, and many factors could have led to heterogeneity, such as the size of MH, different types of surgery, and interval from injury to surgery. However, the complete data were hardly accessed for subgroup analysis, and the factors were relative to treatment decisions. Second, since the studies included were retrospective and prospective observational studies and not RCTs, the comparison between the surgery and observation groups was based on data with a discrepant baseline. Therefore, given the limitations mentioned above, RCTs are needed in the future to evaluate the effectiveness and safety of surgery and observation for TMH. Therefore, we will update our meta-analysis if RCTs are performed in future.

## Conclusions

In conclusion, our systematic review and meta-analysis provides evidence that, compared with observation, surgery leads to higher TMH closure and VA improvement rates. Vitrectomy is an effective and safe treatment method for TMH. The management guidelines for TMH in pediatric patients and the factors affecting the related outcomes need further clarification.

## Data Availability Statement

The original contributions presented in the study are included in the article/Supplementary Material, further inquiries can be directed to the corresponding author.

## Author Contributions

QZ, YX, and HL: conceptualization and design. QZ, HF, and ZF: literature search, data extraction, quality assessment, and statistical analysis. QZ: manuscript writing. HY: supervision. All authors approved the final version of the manuscript.

## Funding

This work was supported by the National Nature Science Foundation of China (Grant Number: 81774371).

## Conflict of Interest

The authors declare that the research was conducted in the absence of any commercial or financial relationships that could be construed as a potential conflict of interest.

## Publisher's Note

All claims expressed in this article are solely those of the authors and do not necessarily represent those of their affiliated organizations, or those of the publisher, the editors and the reviewers. Any product that may be evaluated in this article, or claim that may be made by its manufacturer, is not guaranteed or endorsed by the publisher.
