# Supplementary material for: Vitrectomy vs. Spontaneous Closure for Traumatic Macular Hole: A Systematic Review and Meta-Analysis
Source: Front Med (Lausanne). 2021 Dec 23;8:735968. doi: 10.3389/fmed.2021.735968 (PMC8732763; doi:10.3389/fmed.2021.735968)
Supplement: Supplementary file 1 [file Data_Sheet_1.docx]

Supplementary Material

# List of Captions

| Captions | Contents |
| --- | --- |
| Table 1 | Quality appraisal using the Newcastle-Ottawa Scale in observation group |
| Table 2 | Quality appraisal using the Newcastle-Ottawa Scale in surgery group |
| Figure 1 | Sensitivity analyses |
| Figure 2 | Funnel plots |
| Appendix | Search terms |

# Tables

**Table1 Quality appraisal using the Newcastle-Ottawa Scale in observation group**

| Author, year | Ottawa-Newcastle Scale | | |
| --- | --- | --- | --- |
|  | Selection | Comparability | Exposure |
| Chen H J 2019 | ★★★★ | ★★ | ★★ |
| Fan Y B 2018 | ★★★★ | ★ | ★★ |
| Fu L H 2017 | ★★★★ | ★★ | ★★ |
| Li Y Q 2016 | ★★★★ | ★ | ★★ |
| Yuan L L 2015 | ★★★★ | ★★ | ★★ |
| Chen H Y 2015 | ★★ | ★ | ★★ |
| Tian k 2014 | ★★★★ | ★★ | ★ |
| Hou F 2012 | ★★ | ★ | ★★ |
| Chen S 2010 | ★★★★ | ★ | ★★ |
| Li X W 2008 | ★★ | ★ | ★★ |
| Jin X M 2005 | ★★ | ★ | ★★ |
| YamashitaT 2001 | ★★ | ★ | ★★ |

**Table1 Quality appraisal using the modified Newcastle-Ottawa Scale in surgery group**

| Author, year | Ottawa-Newcastle Scale | | |
| --- | --- | --- | --- |
|  | Selection | Comparability | Exposure |
| Kunikata H 2021 | ★★ | ★ | ★★ |
| Chang J W 2020 | ★★★ | ★★ | ★★ |
| Ghoraba 2019 | ★★★ | ★★ | ★★ |
| Chen H J 2019 | ★★★★ | ★★ | ★★ |
| Fan Y B 2018 | ★★★★ | ★ | ★★ |
| Tang Y F 2018 | ★★ | ★ | ★★ |
| Li D P 2018 | ★★★ | ★★ | ★★ |
| Fu L H 2017 | ★★★ | ★★ | ★★ |
| Li Yan 2017 | ★★★★ | ★★ | ★★ |
| Bor'I A 2017 | ★★ | ★ | ★★ |
| Brennan N 2017 | ★★★ | ★★ | ★ |
| Li Y Q 2016 | ★★ | ★ | ★★ |
| Zhu W K 2016 | ★★★★ | ★ | ★★ |
| Abou Shousha M A 2016 | ★★ | ★ | ★★ |
| Chen S 2015 | ★★ | ★ | ★★ |
| Yuan L L 2015 | ★★ | ★ | ★★ |
| Tian k 2014 | ★★★★ | ★★ | ★★ |
| Hou J 2013 | ★★★★ | ★★ | ★ |
| Wan W C 2013 | ★★ | ★ | ★★ |
| Hammouda H Ghoraba 2012 | ★★ | ★ | ★★ |
| Qu J F 2011 | ★★★ | ★★ | ★★ |
| Ovali T 2010 | ★★ | ★ | ★★ |
| Chen S 2010 | ★★ | ★ | ★★ |
| Gong W 2009 | ★★★★ | ★ | ★★ |
| Wu W C 2007 | ★★ | ★ | ★★ |
| Ma J 2006 | ★★ | ★ | ★★ |
| Liu J J 2004 | ★★ | ★ | ★★ |
| Kuhn F 2001 | ★★ | ★ | ★★ |
| Robert N J 2000 | ★★ | ★ | ★★ |
| García-Arumí J 1997 | ★★ | ★ | ★★ |
| Rubin J S 1995 | ★★ | ★ | ★★ |

# Figure legends

Figure 1. Sensitive analyses. Sensitivity analyses for the TMH closure rate and VA improvement rate. (A) sensitivity analysis for TMH closure rate of surgery group patients; (B) sensitivity analysis for VA improvement rate of surgery group patients; (C) sensitivity analysis for TMH closure rate of observation group patients.

**Figure 2. Funnel plots.** Funnel plots for the TMH closure rate and VA improvement rate. (A) Funnel plot for TMH closure rate of surgery group patients; (B) Funnel plot for VA improvement rate of surgery group patients; (C) Funnel plot for TMH closure rate of observation group patients.


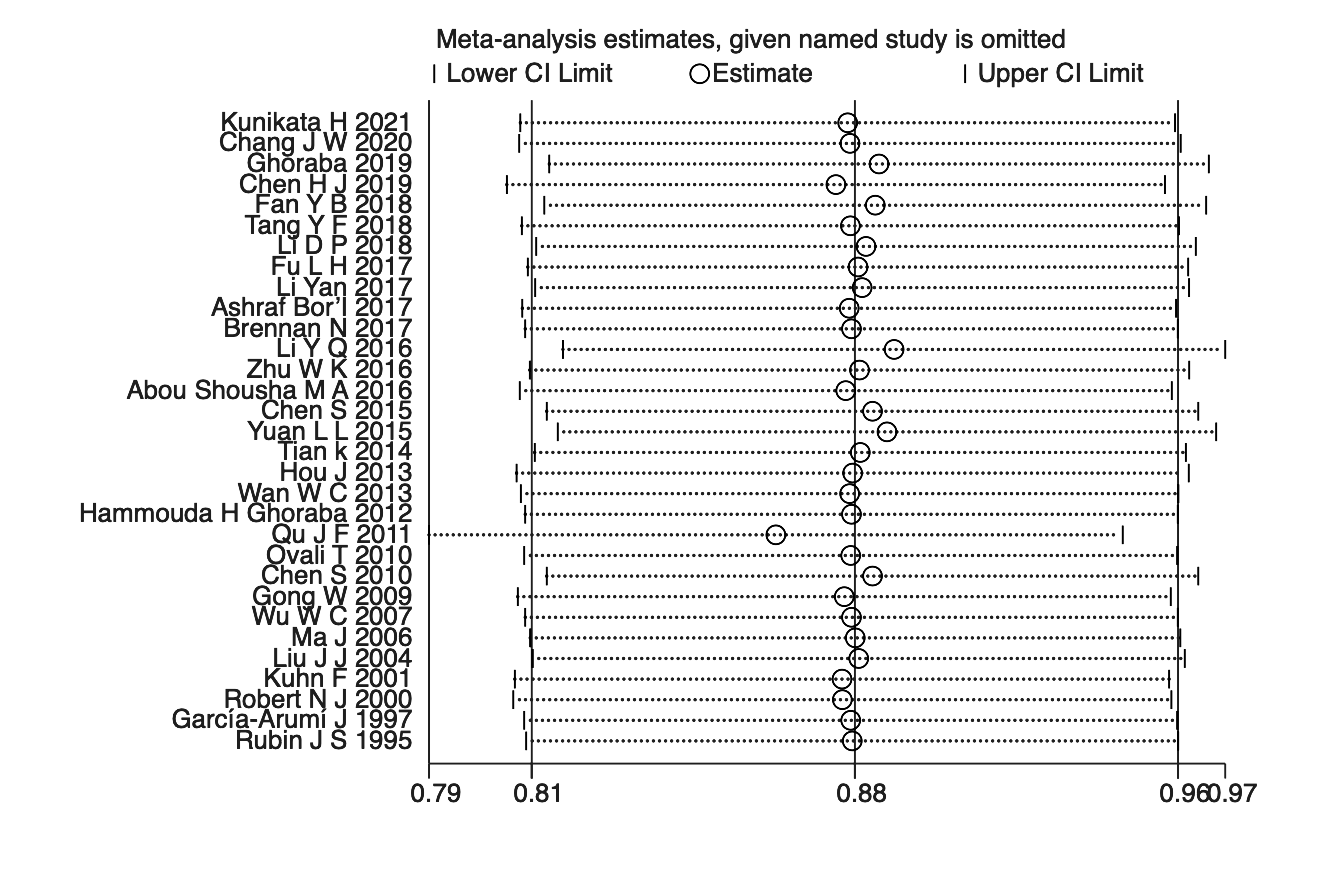


Figure 1A sensitivity analysis for TMH closure rate of surgery group patients


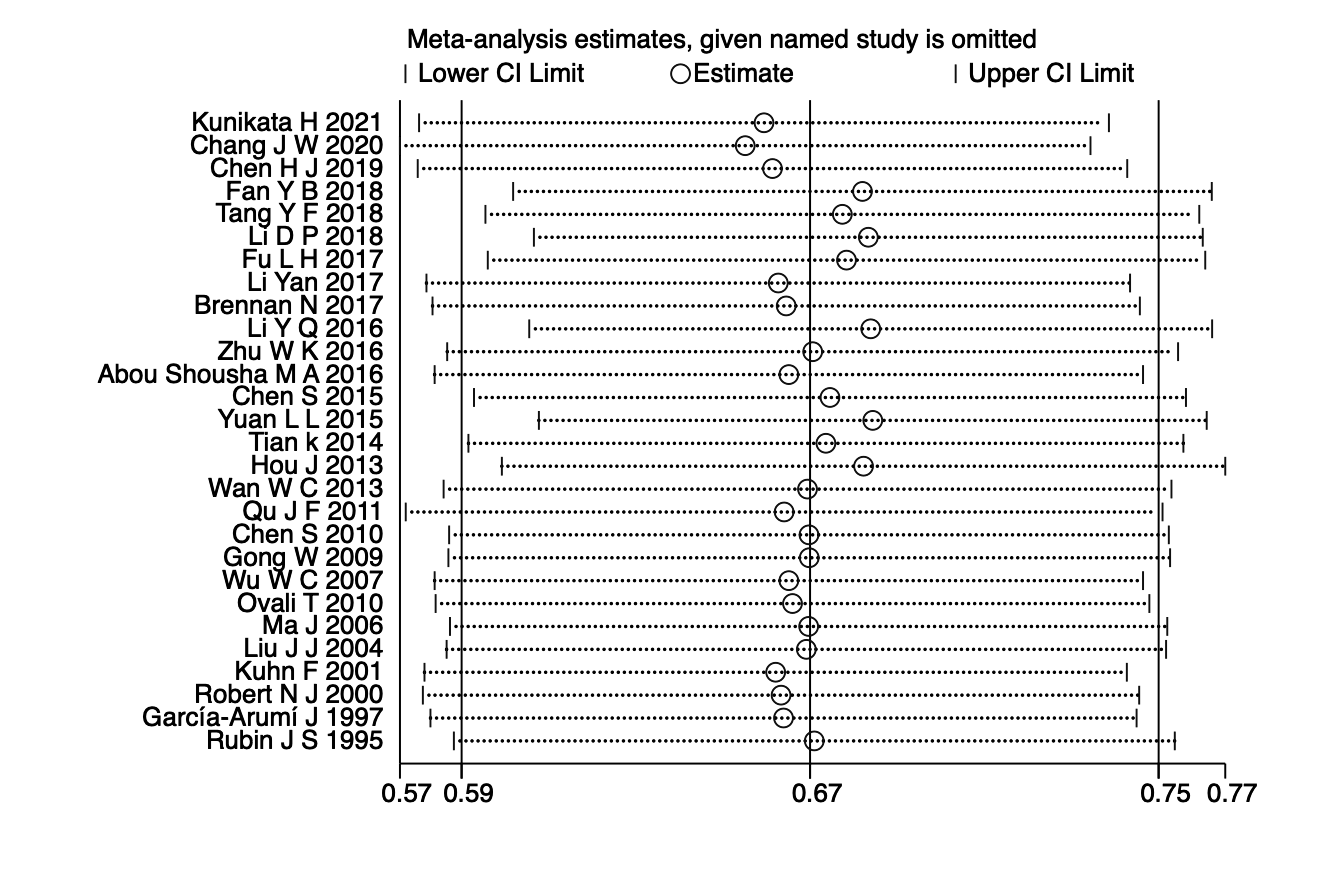


Figure 1B sensitivity analysis for VA improvement rate of surgery group patients


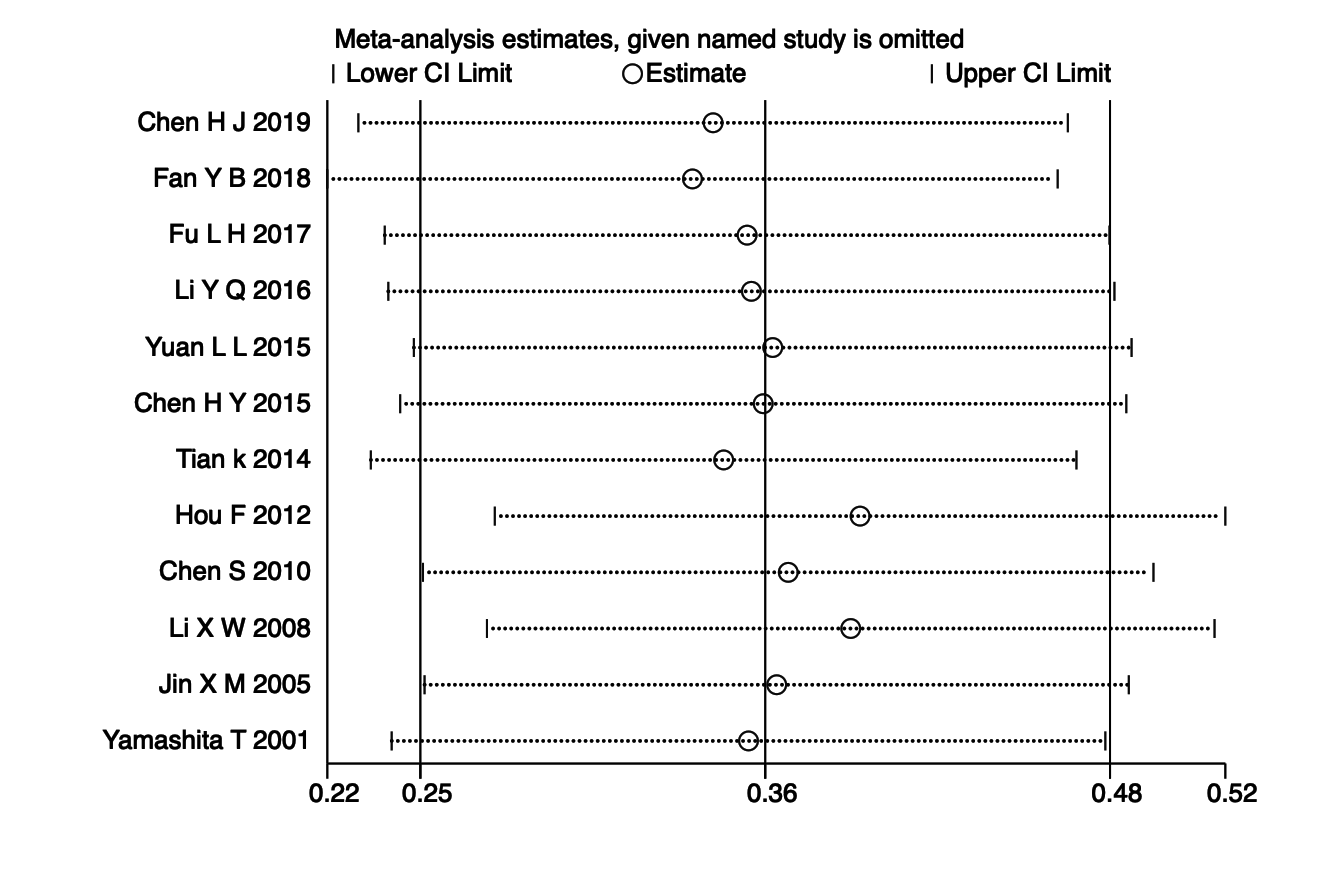


Figure 1C sensitivity analysis for TMH closure rate of observation group patients


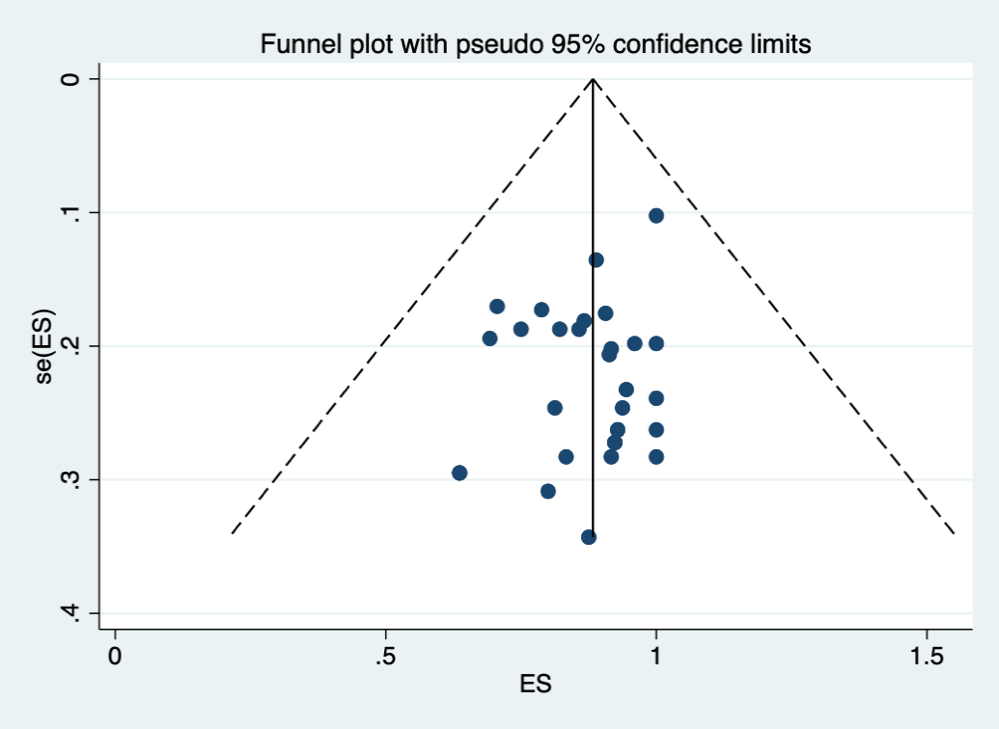


Figure 2A Funnel plot for TMH closure rate of surgery group patients


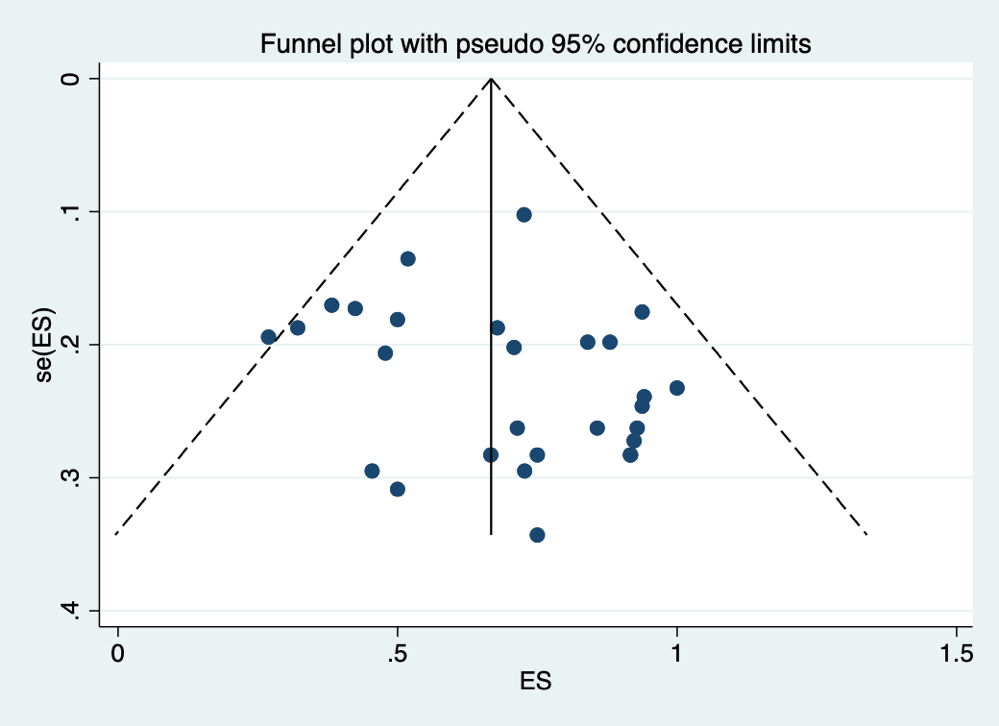


Figure 2B Funnel plot for VA improvement rate of surgery group patients


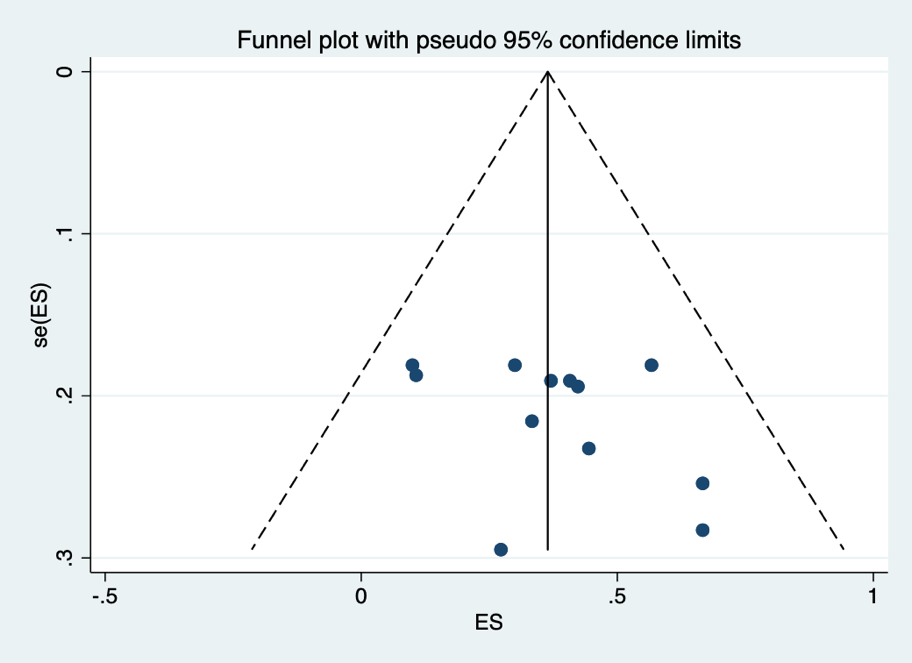


Figure 2C Funnel plot for TMH closure rate of observation group patients

**4 Search terms**

## Appendix Search terms

## Appendix search terms for MEDLINE through PubMed (search data: June 10, 2021)

(((((((pars plana vitrectomy[Title/Abstract]) OR (surgical management[Title/Abstract])) OR ("Vitrectomy"[Mesh])) OR ("Observation"[Mesh])) OR ("Therapeutics"[Mesh])) OR (treatment[Title/Abstract])) AND (((((("Retinal Perforations"[Mesh]) OR (retinal hole[Title/Abstract])) OR (retinal tear[Title/Abstract])) OR (retinal break[Title/Abstract])) OR (macular hole[Title/Abstract])) OR (traumatic macular hole[Title/Abstract]))) AND (((((("Retinal Perforations"[Mesh]) OR (retinal hole[Title/Abstract])) OR (retinal tear[Title/Abstract])) OR (retinal break[Title/Abstract])) OR (macular hole[Title/Abstract])) OR (traumatic macular hole[Title/Abstract]))

**Search terms for Embase (search data: June 10, 2021)**

#1. 'retina tear'/exp

#2. 'retinal perforations':ab,ti OR 'retinal hole':ab,ti OR 'retinal break':ab,ti OR 'macular hole':ab,ti OR 'traumatic macular hole':ab,ti

#3. 'vitrectomy'/exp

#4. 'observation'/exp

#5. 'therapy'/exp

#6. 'surgical management':ab,ti OR 'spontaneous closure':ab,ti OR 'pars plana vitrectomy':ab,ti OR 'treatment':ab,ti

#7. #1 OR #2

#8. #3 OR #4 OR #5 OR #6

#9. #7 AND #8

**Search terms for** **cochrane (search data: June 10, 2021)**

#1 (retinal hole):ab,ti,kw or (retinal tear):ab,ti,kw or (retinal break):ab,ti,kw or (macular hole):ab,ti,kw or (traumatic macular hole):ab,ti,kw

#2 MeSH descriptor: [Retinal Perforations] explode all trees

#3 MeSH descriptor: [Vitrectomy] explode all trees

#4 MeSH descriptor: [Observation] explode all trees

#5 MeSH descriptor: [Therapeutics] explode all trees

#6 (pars plana vitrectomy):ab,ti,kw or (surgical management):ab,ti,kw or (spontaneous closure):ab,ti,kw or (treatment):ab,ti,kw

#7 #1 or #2

#8 #3 or #4 or #5 or #6

#9 #7 and #8

**Search terms for Web of science (search data: June 10, 2021)**

#1 TS=(retinal perforations or retinal hole or retinal tear or retinal break or macular hole or traumatic macular hole)

#2 TS=(vitrectomy or pars plana vitrectomy or surgical management or observation or spontaneous closure or Therapeutics or treatment)

#3 #1 AND #2
